# Design, synthesis, and characterization of novel system x_C_^−^ transport inhibitors: inhibition of microglial glutamate release and neurotoxicity

**DOI:** 10.1186/s12974-023-02972-x

**Published:** 2023-12-06

**Authors:** Mariusz P. Gajewski, Steven W. Barger

**Affiliations:** 1 Department of Physical and Earth Sciences, Arkansas Tech University, McEver Building, 1701 N Boulder Ave, Russellville, AR 72801 USA; 2 Departments of Geriatrics and Neurobiology and Developmental Sciences, University of Arkansas for Medical Sciences, Little Rock, AR USA; 3 Geriatric Research Education and Clinical Center, Central Arkansas Veterans Healthcare System, Little Rock, AR USA

**Keywords:** Amino acid transport system xc protein, Excitotoxicity, Cell death, Drug discovery, Glutamic acid, Microglia, Stress, Oxidative

## Abstract

Neuroinflammation appears to involve some degree of excitotoxicity promulgated by microglia, which release glutamate via the system x_C_^−^ (Sx_C_^−^) cystine-glutamate antiporter. With the aim of mitigating this source of neuronal stress and toxicity, we have developed a panel of inhibitors of the Sx_C_^−^ antiporter. The compounds were based on l-tyrosine, as elements of its structure align with those of glutamate, a primary physiological substrate of the Sx_C_^−^ antiporter. In addition to 3,5-dibromotyrosine, ten compounds were synthesized via amidation of that parent molecule with a selection of acyl halides. These agents were tested for the ability to inhibit release of glutamate from microglia activated with lipopolysaccharide (LPS), an activity exhibited by eight of the compounds. To confirm that the compounds were inhibitors of Sx_C_^−^, two of them were further tested for the ability to inhibit cystine uptake. Finally, these agents were shown to protect primary cortical neurons from the toxicity exhibited by activated microglia. These agents may hold promise in reducing the neurodegenerative effects of neuroinflammation in conditions, such as encephalitis, traumatic brain injury, stroke, or neurodegenerative diseases.

## Introduction

System x_C_^−^ (Sx_C_^−^) is an obligate-exchange transport protein which expels glutamate (Glu) and imports cystine (Cys)_2_ through the plasma membrane [1]. This protein complex is localized in many cell types and invoked in several biological processes inside and outside of the CNS. Some of these processes contribute to pathological outcomes, particularly in the CNS, where the transporter’s capacity to release Glu can contribute to excitoxicity [2–7]. Thus, the development of pharmacological agents which can inhibt Sx_C_^−^ may be of therapeutic benefit.

One pathology-related Sx_C_^−^ phenomenon is release of Glu from microglia activated under conditions of neuroinflammation. Activated microglia produce abundant superoxide, consuming the cell’s major antioxidant: glutathione. While some fraction of glutathione is merely oxidized and is thus available for reuse after action by glutathione reductase, detoxification of oxidized lipids requires a covalent adduct of glutathione, thus depleting glutathione irreversibly [5, 8]. Replenishment of glutathione via de novo synthesis requires cysteine (Cys), which creates a Cys sink, quenched by (Cys)_2_ import. Because Sx_C_^−^ is the major mediator of (Cys)_2_ import, microglia release Glu into the extracellular space as (Cys)_2_ follows this concentration gradient. The role of Sx_C_^−^ in this phenomenon is indicated by its attenuation in the absence of extracellular (Cys)_2_ or in the presence of α-aminoadipic acid or sulfasalazine [2–4, 7, 9]. The release of Glu from this extrasynaptic source may be particularly accessible to extrasynaptic Glu receptors, which have an especially important role in excitotoxicity [10]. Considerable evidence indicates that conversion of oxidative stress into an excitotoxic stress by the chain of events that engage Sx_C_^−^ has a substantial neurological impact [5, 7, 11–13]. A harmful release of Glu via Sx_C_^−^ may arise in any pathological condition that includes the activation of microglia, including microbial infections, trauma, stroke, and chronic degenerative disorders; evidence of a role for Sx_C_^−^ has been documented in amyotrophic lateral sclerosis [7]. Although α-aminoadipic acid and several other compounds are effective at inhibiting Sx_C_^−^ in vitro (for review see [1]), currently available agents have off-target effects, instability, or other attributes that limit their drug-likeness.

We developed a number of Sx_C_^−^ inhibitors and demonstrated and tested their actions against Sx_C_^−^ activity. Because microglial release of Glu via Sx_C_^−^ is both an index of the antiporter’s activity and a potential contributor to neuroinflammatory damage, we exploited this paradigm as a bioassay. We also carried two of these compounds forward in tests against neurotoxicity. Results indicate that analogs based on the structures discussed here could inhibit Sx_C_^−^ activity and potentially ameliorate the neuronal damage associated with CNS inflammation.

## Methods

### Drug design

De novo design of potential inhibitors of Sx_C_^−^ utilized computational methods, namely, SYBYL 8.0 and Spartan’16 packages. The proposed pharmacophore was based on the structure and physicochemical properties of the endogenous transportable substrate Glu, exploiting the locked conformer inherent in l-tyrosine. Computational pharmacokinetics studies were powered by SwissADME model and besides bioavailability, the absorption and blood–brain barrier penetration properties were analyzed and graphed applying the *Brain Or IntestinaL EstimateD permeation* (BOILED-Egg) framework.

### Synthesis

**Fig. 1:**
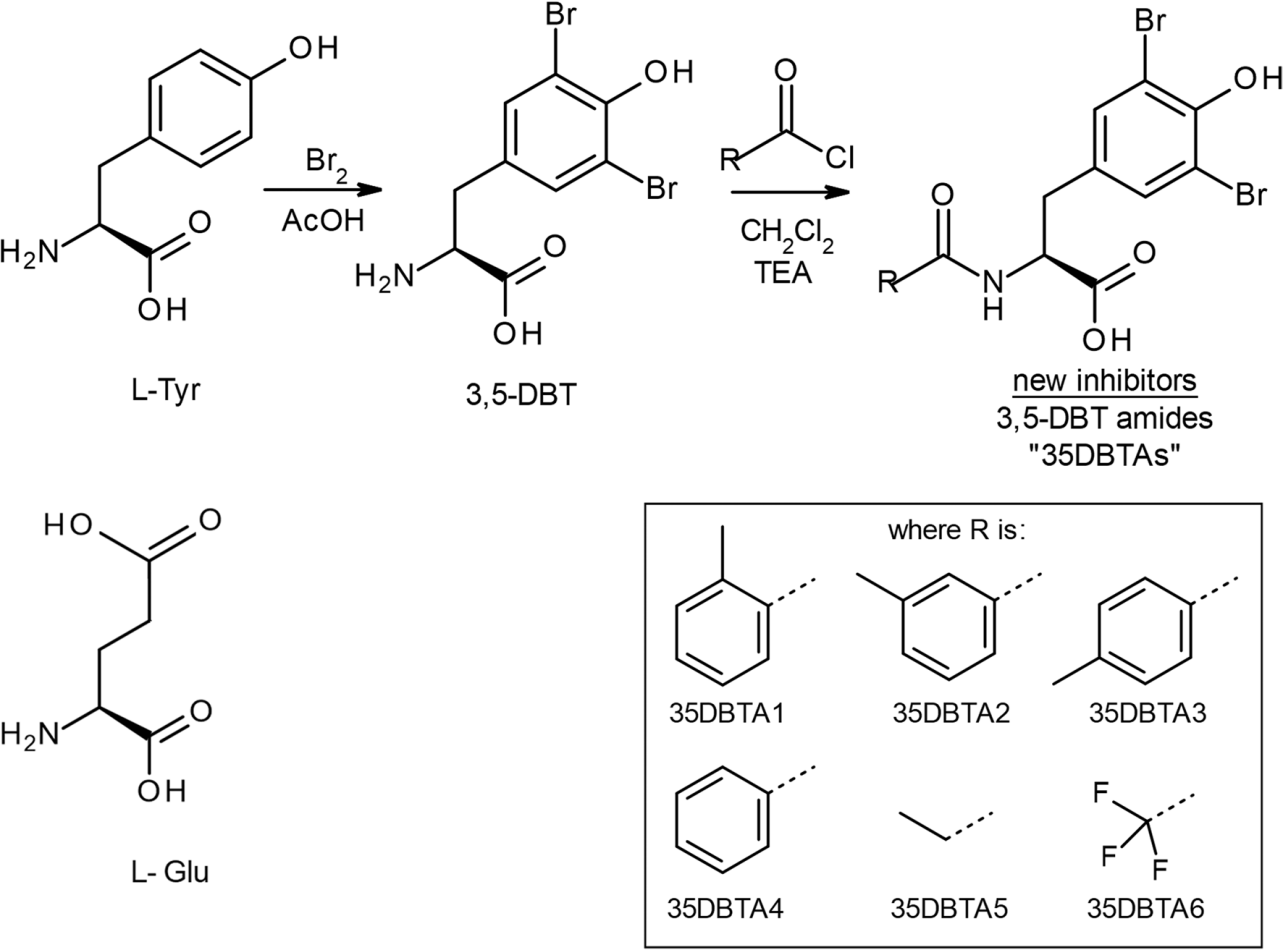
Design and scaffold of the first generation of prospective Sx_C_^−^ inhibitors. l-Tyr reflects the shape of a locked conformer of l-Glu but has no activity at Sx_C_^−^. Halogenation of the aromatic ring of Tyr, followed by amidation of the molecule with a selection of acyl halides at 3,5-DBT's α-amino group allowed several chemical structures to be tested, varying in a branch of the scaffold hypothesized to interact with a hydrophobic pocket in the transporter

Potential inhibitors of the Sx_C_^−^ transporter were synthesized by means of standard solution phase organic synthesis. Most of these were produced by electrophilic aromatic substitution/bromination of the aromatic ring of l-tyrosine (Tyr) to form 3,5-dibromotyrosine (3,5-DBT). This was followed by amidation of the α-amino group of 3,5-DBT with a selection of acyl halides. The latter was accomplished by adopting standard Schotten–Baumann approach. The synthesis leading to these molecules is outlined in Fig. 1. A 3,5-diiodo derivative of tyrosine (3,5-DIT) was also tested, as was 2,5-diiodohistidine (2,5-DIH).

### Characterization

TLC of the products indicated the presence of contaminants. These were removed by dissolving the products in a diluted NaOH aqueous solution, followed by precipitation with 6 N HCl, twice. The precipitated products were dried, redissolved in ethyl acetate and resolved by chromatography on a preparatory silica plate in 5:5:1 ethyl acetate:hexane:methanol. The standard spectroscopic methods (MS, IR, ^1^H and ^13^C-NMR) were used to characterize the compounds before the focus was shifted to bioassays.

### Glutamate release assay

Primary cultures of rat microglia were established as described previously [14]. Microglia were seeded at 375,000/cm^2^ in 96-well plates with minimal essential medium with Earle’s salts (MEM) containing 10% fetal bovine serum (FBS). After an overnight plating, the cultures were washed twice with serum-free MEM, and then a third volume was applied containing test substances at the indicated concentrations; MEM contains 100 μM (Cys)_2_. Immediately thereafter, lipopolysaccharide (LPS) was added to some wells at 100 ng/ml. This concentration was selected for this particular lot of LPS as the concentration producing a slightly submaximal response (i.e., 2–5% below the maximum). After 18 h, 50 μl of medium was transferred from each well to a black, opaque-bottomed 96-well plate and combined with 50 μl of assay reagent from a Amplex® Red Glutamic Acid Assay kit (ThermoFisher). A standard curve was generated with a range of concentrations of l-glutamate (monosodium salt). The assay reagent contained 0.25 U/ml horseradish peroxidase, 0.08 U/ml l-glutamate oxidase, 0.5 U/ml l-glutamate/pyruvate transaminase, and 200 μM l-alanine. After 30 min, fluorescence was measured in a Molecular Devices SpectraMax 3 with excitation at 550 nm and emission at 590 nm; values were interpolated into the standard curve. The microglial medium was also assayed for nitric oxide using Griess reagent to confirm that none of the experimental compounds was a general inhibitor of microglial activation. We also considered whether any compound was toxic to microglia. The microglia remaining in the 96-well plates were combined with 3-(4,5-dimethylthiazol-2-yl)-2,5-diphenyl-2*H*-tetrazolium bromide (MTT) at a final concentration of 125 μg/ml (in MEM/FBS) and incubated for 30 min at 37 °C and 5.5% CO_2_. After removal of the medium, 100 μl of dimethyl sulfoxide (DMSO) was added to dissolve the formazan crystals, and absorbance at 540 nm was measured in the SpectraMax 3; the mean background obtained in wells containing DMSO alone was subtracted from each experimental value.

### Cystine uptake assay

Cystine uptake was measured essentially as per Shimomura et al. [15] using the Cystine Uptake Assay Kit (DOJINDO). Primary microglia were seeded as per glutamate release assays, and some cultures were treated the following day with 100 ng/ml LPS. After 15 h, the cultures were washed twice with Hank’s balanced salt solution (HBSS) and then incubated 5 min at 37 °C in HBSS containing fresh LPS and test substances at the indicated concentrations. The cultures were then exposed to HBSS containing selenocystine (DOJINDO) for 30 min at 37 °C, after which they were washed three times with ice-cold phosphate-buffered saline and then fixed in 50 μl methanol. A detection buffer comprising fluorescein *O*,*O*’-diacrylate and reducing agent (DOJINDO) was then added, and the plate was sealed and incubated for 30 min at 37 °C. Reaction product was then assayed in the Molecular Devices SpectraMax 3 with excitation at 490 nm and emission at 535 nm. Blanks consisted of wells containing only cells that had been washed, fixed with methanol, and incubated with the detection buffer, and the values from these wells were subtracted from the experimental values.

### Neurotoxicity assay

Primary cultures of rat cortical neurons were established as described previously [16]; incorporation of both a serum-free medium and an intermittent treatment with cytosine arabinoside results in cultures that are among the purest neuronal primary cultures available. The cultures were seeded in 24-well plates, and 8 days later primary microglia were plated in transwell inserts (12-mm diameter) at 375,000/cm^2^, washed to serum-free MEM, and treated with test substances followed immediately by LPS (100 ng/ml). After 1 h, the inserts were placed into the wells of the neuronal cultures. After an additional 18 h, the inserts were removed, and neuronal survival was evaluated by MTT assay as described above. Values are represented relative to the MTT signal produced in cultures exposed to untreated microglia.

### Statistical analysis

Data were analyzed in GraphPad Prism 9.5.1. Nonlinear regression was performed on the glutamate release assay data to derive IC_50_ values and the graphed plot lines. ANOVA indicated post hoc tests were applied to dosimetric data; *P* < 0.05 was considered significant.

## Results

The drug-design strategy was based on the structure of the fundamental, endogenous, transportable substrate Glu. Tyr was identified as a convenient scaffold molecule; 3D molecular modeling revealed a close similarity between Glu and Tyr, with the exception of the p*K*_a_ of the distal carboxy group in Glu and the phenolic acidic proton in Tyr (~ 5 orders of magnitude difference in acidity). However, initial assays demonstrated that Tyr does not possess any inhibitory properties on the Sx_C_^−^ transporter, despite being a close mimic of Glu. Attention was then focused on increasing the acidity of the phenolic proton in Tyr, which led to the development of 3,5-dibromotyrosine (3,5-DBT) (Fig. 1). This compound was the first tyrosine-based Sx_C_^−^ inhibitor capable of potent inhibition of Sx_C_^−^ transport, and 3,5-DBT was used as a bioactivity reference molecule in subsequent assays of Sx_C_^−^ activity. Even though the molecule is easy to synthesize and convenient in use (high activity, high solubility in water as a salt, and easily detected by MS due to presence of two bromine atoms), it is also very polar. Computational methods (SwissADME) and fundamental knowledge led to concerns that 3,5-DBT would be far too polar to cross the blood–brain barrier (BBB). Thus, efforts were focused on improving the pharmacodynamics through further modification of the amino acid. Previous structure–activity relationship (SAR) studies indicated that Sx_C_^−^ transporter has a large, lipophilic pocket that interacts with the α-amino side of 3,5-DBT. Prior experiments indicated that this amino group is likely dispensible for the compound’s inhibitory activity, so it may be considered an excellent attachment point to be exploited in subsequent lead optimization steps. This strategy proved very useful in manipulation of the pharmacokinetic properties of the new molecules until desired parameters were obtained. Several new 3,5-DBT analogs were prepared as 3,5-DBT α-amino amide derivatives (“35DBTA#”; Fig. 1).

**Fig. 2:**
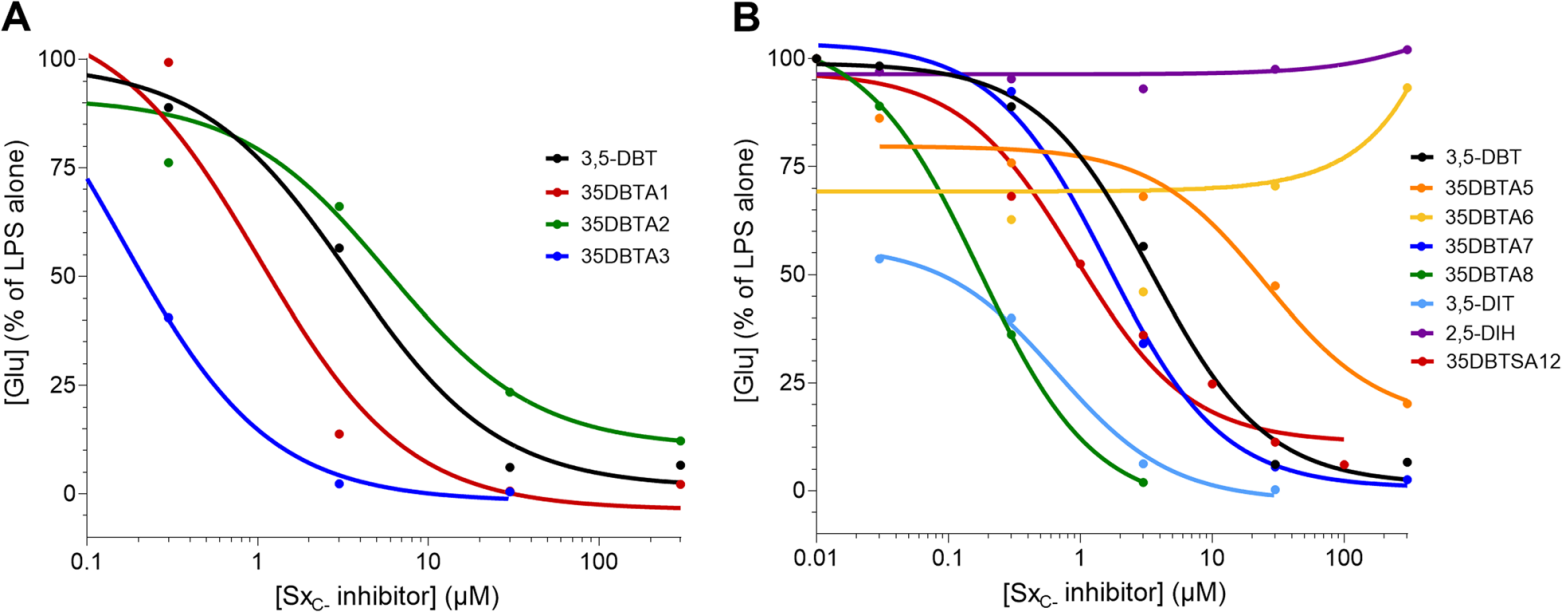
Microglial Glu release assay. Rat primary microglia were activated with 100 ng/ml LPS alone or immediately following application of the indicated concentrations of test substances. After 18 h in the presence of LPS, an aliquot of medium was assayed for Glu via a glutamate dehydrogenase colorimetric assay. **A** First generation of compounds was created by simply varying the position of a methyl group around an aromatic ring, comparing activity to 3,5-DBT. **B** Additional modifications were evaluated, including several that simply extend the size of the structure hypothesized to fit into the hydrophobic pocket of the Sx_C_^−^ transporter. Values are represented relative to the Glu detected in cultures treated with LPS alone; each data point represents a mean of 4 cultures. Nonlinear regression was performed to provide the plot lines

The primary objective of developing Sx_C_^−^ transport inhibitors was to block the release of glutamate from activated microglia. This parameter has been shown to be a reliable measure of Sx_C_^−^ activity and useful tool in screening for its inhibitors [17]. We assayed glutamate levels in the culture media of primary microglia exposed to LPS for 18 h (Fig. 2). While LPS is not a component of most naturally occurring instances of neuroinflammation, it shares with other, more widely relevant stimuli the ability to activate Sx_C_^−^. Initial screening focused on structures, wherein an aromatic ring was attached to the amide site, with a methyl group in various positions on the aromatic ring. Promising activity was observed with 35DBTA3, which has the methyl group in the *para*-position (Fig. 1). The *ortho-* and *meta-*analogs were effective, but 35DBTA3 displayed the greatest potency (Fig. 2A).

**Fig. 3:**
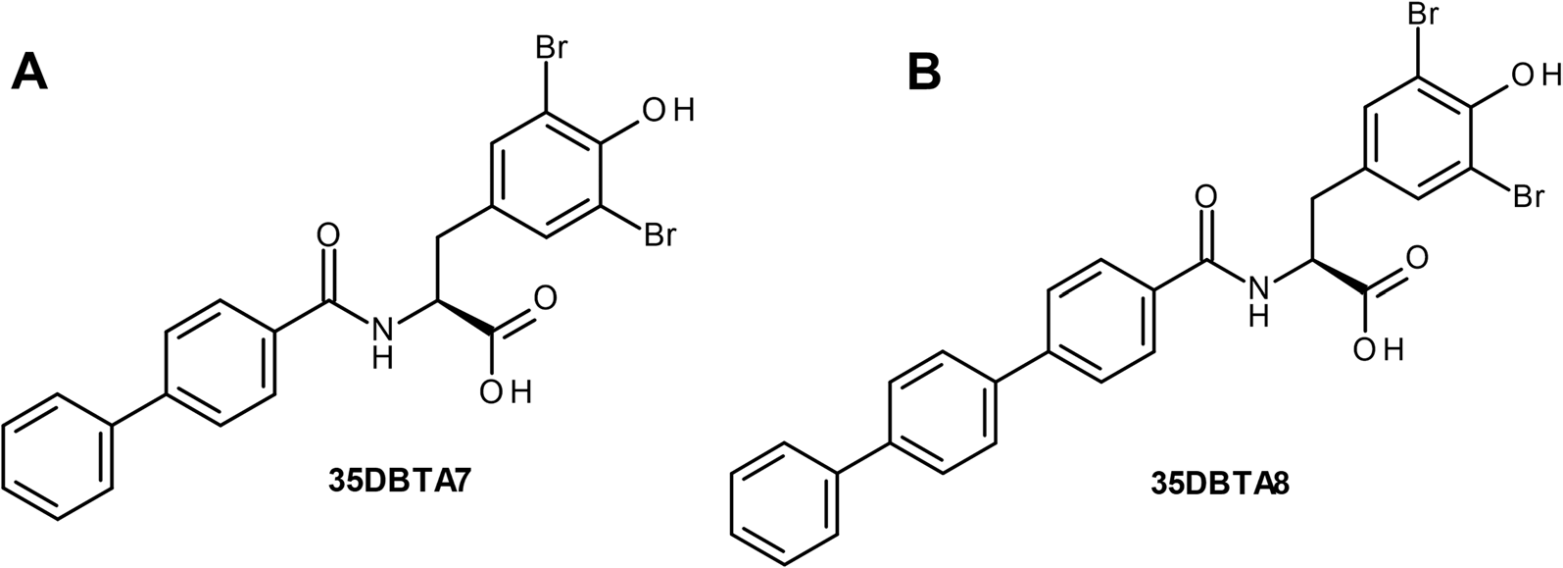
Structures of 35DBTA7 and 35DBTA8

**Fig. 4:**
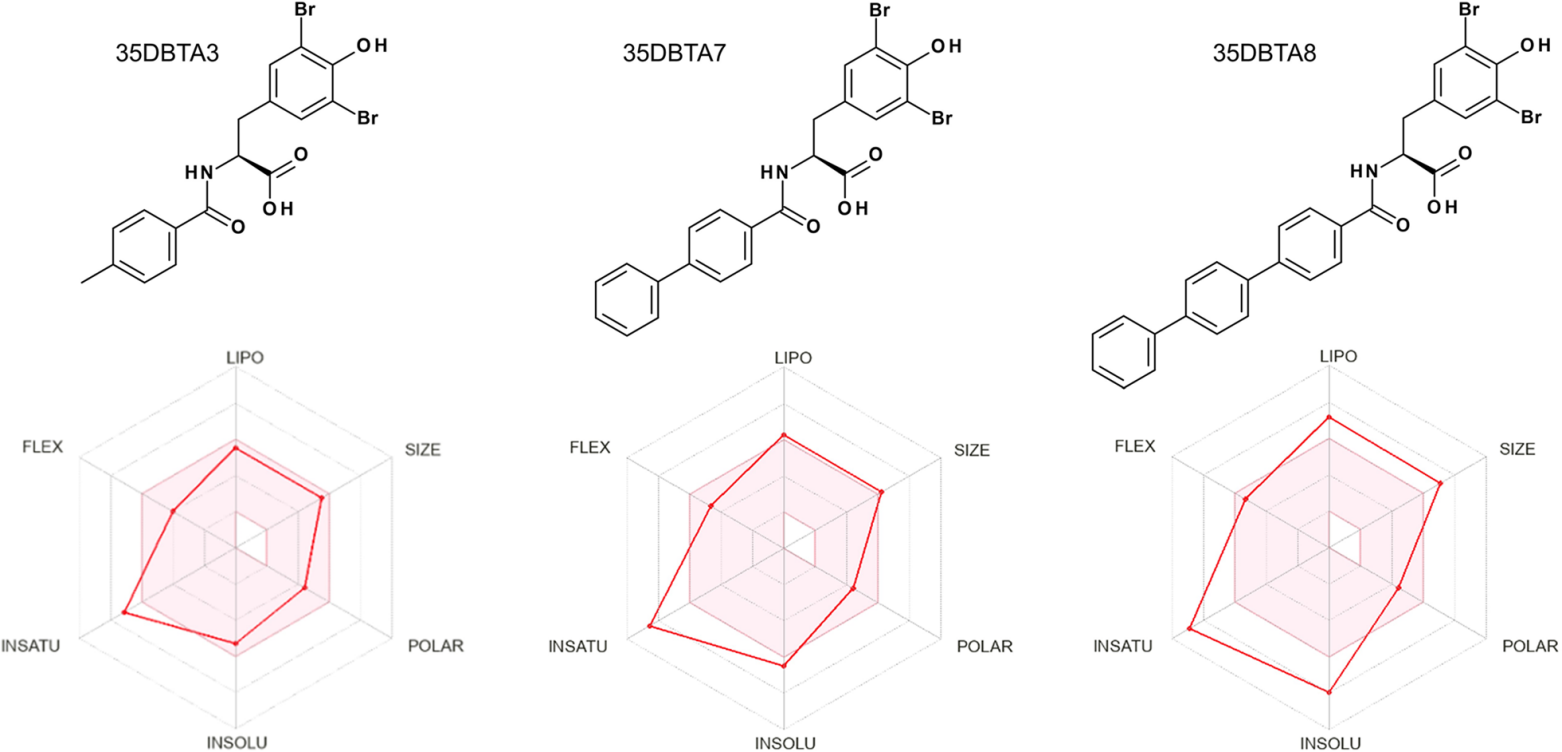
Drug-likeness and bioavailability of 35DBTA3, 35DBTA7 and 35DBTA8

It remained possible that the methyl group at the *para-*position of 35DBTA3 was not necessarily providing the optimal interaction with the transporter’s lipophilic pocket. To further explore the architecture of the Sx_C_^−^ pharmacophore for optimizing the lead compound, another inhibitor was designed, where an extension of the amide moiety by another aromatic ring was introduced. The simplest of these constructs was 35DBTA7 (Fig. 3A). Glu efflux inhibition experiments demonstrated efficacy for 35DBTA7 with an IC_50_ = 1.615 μM (Fig. 2B). Following same strategy, further extension of the amide side substituent with an additional aromatic ring produced 35DBTA8 (Fig. 3B). This new compound proved to be the most potent inhibitor in this series (IC_50_ = 183.5 nM concentration; Fig. 2B). However, the increased hydrophobicity of this molecule made it poorly soluble in aqueous solutions, rendering it impractical (Fig. 4).

Amides of 3,5-DBT are vulnerable to hydrolysis at the amide bond. Therefore, a new molecule was designed: 35DBTSA12, a sulfonamide analog of 35DBTA3, which was anticipated to be more stable. This compound exhibited an IC_50_ of 1.16 μM, similar to that of 35DBTA7 (Fig. 2B). To further explore the potential for amino acid derivatives to modulate Sx_C_^−^, we also tested 3,5-DIT and 2,5-DIH. While the latter was completely inactive, 3,5-DIT was approximately an order of magnitude more potent that 3,5-DBT (Fig. 2B). The IC_50_ values for all the compounds tested in the Glu-release assay are provided in Table 1.

**Table 1:** IC_50_ values of Sx_C_- inhibitors

Compound	IC_50_ ± SEM (μM)
3,5-DBT	3.490 ± 0.7788
35DBTA1	1.028 ± 0.4401
35DBTA2	5.793 ± 3.96
35DBTA3	0.1587 ± 0.0244
35DBTA5	25.48 ± 4.59
35DBTA6	N.D
35DBTA7	1.615 ± 0.3766
35DBTA8	0.1720 ± 0.0056
3,5-DIT	0.6647 ± 0.1265
2,5-DIH	N.D
35DBTSA12	0.9081 ± 0.3106

Nonlinear regressions were performed in GraphPad Prism, which also provided IC_50_ ± SEM

**Fig. 5:**
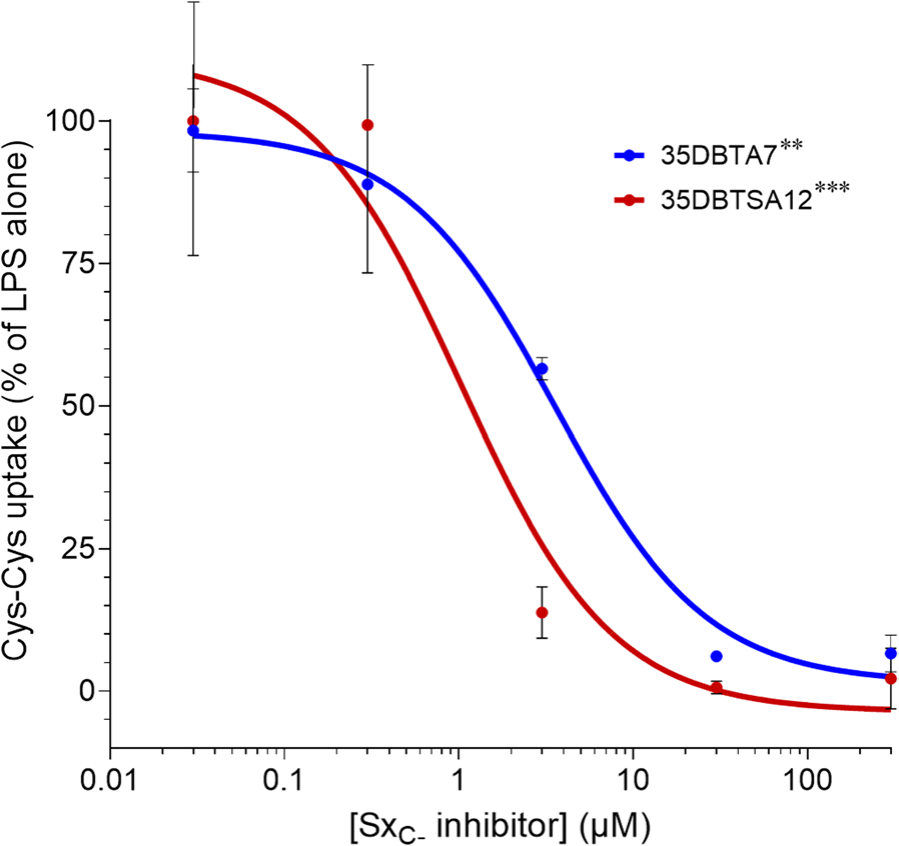
35DPTA7 and 35DPTSA12 inhibit cystine uptake in microglia. Rat primary microglia were activated with 100 ng/ml LPS alone for 15 h, then the indicated concentrations of test substances were applied during application of selenocystine. After 30 min, uptake was stopped and assayed fluorometrically. Values are mean ± SEM of quadruplicate cultures. ****P < 0.0001 for all concentrations of 35DBTA7 vs. LPS alone; **P = 0.012 for 0.03 μM vs. 300 μM 35DBTA7, ***P = 0.0009 for 0.03 μM vs. 300 μM 35DBTSA12 (ANOVA and Bonferroni post hoc)

Glutamate may be released from microglia by mechanisms other than Sx_C_^−^ [18]. To confirm that Sx_C_^−^ was the transporter inhibited by the novel DBT analogs, cystine uptake was measured for two of the test substances. Primary microglia were treated for 15 h, and then 35DBTA7 and 35DBTSA12 were applied during uptake of selenocystine, which can be detected after conjugation with fluorescein *O*,*O*’-diacrylate (Fig. 5). A dose-dependent inhibition of cystine uptake into microglia was observed for both compounds at IC_50_ values roughly similar to those for glutamate release (Fig. 2, Table 1).

**Fig. 6:**
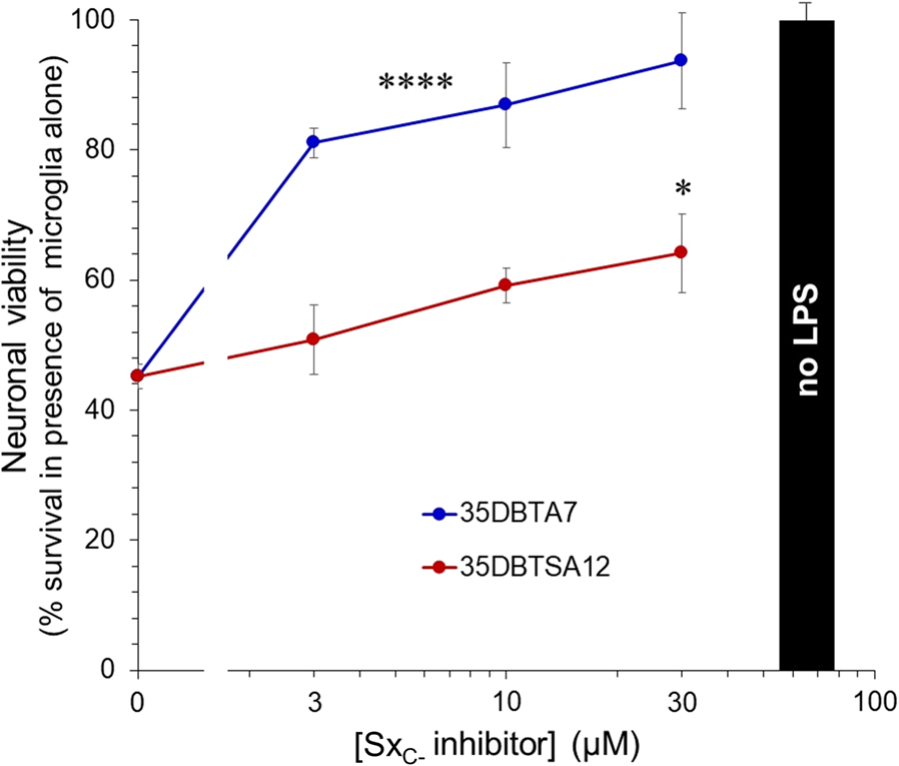
35DPTA7 and 35DPTSA12 reduced the neurotoxicity of activated microglia. Primary microglia were plated on permeable membranes in basket-type cell-culture inserts. Some cultures were treated with the indicated concentrations of 35DBTA7 or 35DBTSA12, followed immediately by application of LPS (100 ng/ml); some cultures were untreated and some received LPS alone. After 1 h, the inserts were placed into wells containing primary cortical neurons. After an additional 18 h, neuronal survival was determined by MTT assay. Values represent the percentage of the values obtained in wells containing untreated microglia (black bar); the y-intercept represents the neuronal viability values obtained in the presence of microglia treated with LPS alone. Values are mean ± SEM of quadruplicate cultures. ****P < 0.0001 for all concentrations of 35DBTA7 vs. LPS alone; *P = 0.039 for 30 μM 35DBTSA12 vs. LPS alone (ANOVA and Dunnett post hoc)

The ultimate objective of development of Sx_C_^−^ transport inhibitors for neuroinflammatory conditions is to reduce neurotoxicity under these conditions. To test the potential for such neuroprotection, 35DBTA7 and 35DBTSA12 were screened in a coculture model that allows agents secreted by microglia to diffuse to neurons without contact between the microglia and neurons. A considerable fraction of neurotoxicity found in microglial conditioned medium can be blocked with glutamate receptor antagonists, particularly those of the NMDA class: 2(*R*)-2-amino-5-phosphonopentanoic acid; 5,7-dicholorokynurenic; and MK-801 [2, 4, 19, 20]. Primary microglia plated in transwell inserts were activated by LPS in the presence or absence of prospective Sx_C_^−^ inhibitors, then the microglial transwells were transferred to culture wells containing primary cortical neurons. Relative neuronal survival was determined 18 h later via MTT assay. Both 35DBTA7 and 35DBTSA12 elevated the survival signal (Fig. 6), likely through their documented suppression of glutamate release from the microglia. Despite the similar IC_50_ values for these two compounds in microglial release assays, 35DBTA7 was considerably more effective in neuroprotection than 35DBTSA12.

## Discussion

Microglia play important roles in neutralizing infectious pathogens, responding to traumatic or ischemic damage, and shaping synaptic/dendritic elements under conditions of neuroplasticity. However, excessive activation of microglia under conditions generally characterized as neuroinflammation can contribute to neurodegeneration [21]. Though neuroinflammation is a complex phenomenon associated with release of cytokines and other factors, a potent component of their neurotoxicity is the release of excitatory amino acid transmitters through Sx_C_^−^ [2, 11, 22, 23]. Therefore, we have pursued the development of chemical agents that can inhibit Sx_C_^−^ transport, anticipating neuroprotection during conditions of neuroinflammation. Approximately, a dozen compounds were designed, synthesized, and tested. LPS was used to activate microglia and induce their release of glutamate and uptake of cystine via Sx_C_^−^. While microglia have been reported to release glutamate via connexin hemichannels [18], this mechanism does not appear to contribute to the glutamate release under the conditions reported here, which is sensitive to α-aminoadipic acid, lipophilic antioxidants, protein kinase A inhibition, and removal of cystine from the medium [5, 14]. Moreover, two members of the novel compounds were confirmed to inhibit cystine uptake. The analysis of 35DBTA7 and 35DBTSA12 was later extended to assays of neuroprotection in the face of activated microglia. Both compounds displayed their ability to reduce microglial release of excitotoxic levels of Glu in vitro.

Our early structure–activity relationship (SAR) studies implied that there is a large lipophilic pocket in the Sx_C_^−^ protein on the side of the α-amino substituent of the inhibitors. After identification of the first inhibitor lead (3,5-DBT), a panel of new molecules for SAR studies was designed and prepared. Remarkable activity of 35DBTA3 (as compared to 35DBTA1, 35DBTA2, and 35DBTA4) drew the attention to the importance of the lipophilic substituent in the *para-*position of the aromatic system of the amide part. That position was later thoroughly explored. The new molecules, designated 35DBTA7 and 35DBTA8 were quite effective at inhibiting microglial glutamate release. It is important to point out that, besides the pharmacokinetics, one of the key factors guiding us in selection of promising inhibitors is their action at submicromolar concentrations. One inhibitor that did not follow trends was 35DBTA6, a trifluoroacetamide of 3,5-DBT. This molecule seemed to trend toward inhibitory properties at low concentrations but produced a reversal of that trend above 3 μM. Other agents that cause cell death elevate extracellular Glu, presumably due to escape from the cytosol of lysed cells. Thus, the biphasic nature of 35DBTA6 may reflect toxicity at higher concentrations, a common finding with fluorine derivatives. Because one of the concerns was the possibility of premature hydrolysis of the new inhibitors (the amide bond), a stable sulfonamide 35DBTSA12 (a direct sulfo analog of 35DBTA3) was designed and tested. Even though its activity was similar to that of 35DBTA7, it showed diminished potency in the neuroprotection assay. 3,5-DIT displayed promising potency but proved to be exceedingly light sensitive and, therefore, less appealing.

To extend the relevance of the in vitro screening, the two compounds were tested in an assay of neuroprotection. Although other factors have been identified that mediate neurotoxicity in neuroinflammation, we find that Glu receptor agonists are among the most potent. For instance, neuroprotection in a microglia–neuron coculture was far superior with an inhibitor of nitric oxide synthase (NOS) 1—the neuronal isoform activated by Glu receptor agonism—than with an inhibitor of NOS2—the isoform expressed in activated microglia [4]. It is possible that Glu released via Sx_C_^−^ is potentiated by other microglia-derived Glu receptor ligands, such as D-serine or quinolinic acid [24, 25]. In addition, cytokines, reactive oxygen species, and proteases may play roles over distances in space and time [21]. Nevertheless, we and others find that a substantial fraction of microglial neurotoxicity can be alleviated by blocking the effects of Glu. Here, we found that 35DBTA7 was quite effective in such a model of indirect neurotoxicity. The lower efficacy of 35DBTSA12 may indicate that it exerts a mild degree of direct neurotoxicity of its own. Alternatively, it is possible that 35DBTA7 actually served as a prodrug, hydrolyzing to 3,5-DBT for greatest efficacy. If so, this relationship might be exploited in vivo, for instance, to delay the formation of 3,5-DBT until the prodrug has crossed the blood–brain barrier, thus trapping and concentrating the effective agent in the CNS.

**Fig. 7:**
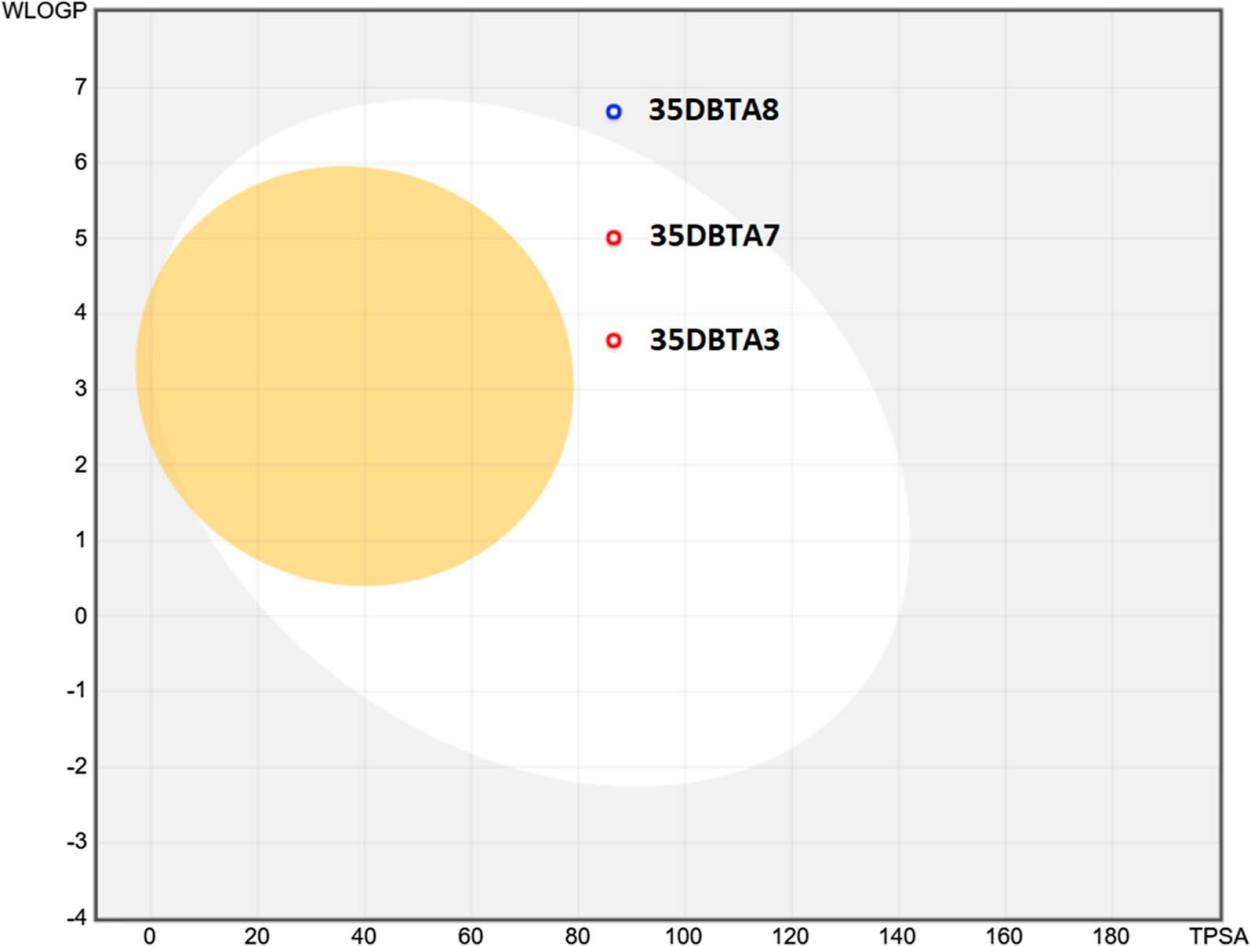
Brain Or IntestinaL EstimateD permeation method (BOILED-Egg) model portrays 35DBTA8 as a poor candidate for a drug that would partition to the CNS

Because of their remarkable potency, 35DBTA3 and 35DBTA8 seem worth investigating further. However, 35DBTA8, being the best inhibitor from an activity standpoint, is computationally classified as having poor bioavailability, possibly not crossing the GI tract membrane (Fig. 4). In addition, the *Brain Or IntestinaL EstimateD* (BOILED-Egg) permeation model portrays 35DBTA8 as a poor candidate for a drug that would partition to the CNS (Fig. 7). Relatedly, we encountered problems with solubility of this compound in aqueous media; even its disodium salt (carboxylate and phenoxide) was poorly soluble, producing turbid solutions/suspensions. This rendered 35DBTA8 impractical for our applications and narrowed the choices down to 35DBTA7 and 35DBTSA12. It may be worth noting that very potent inhibitors such as 35DBTA3 can have drawbacks, including effects that approach a binary “on/off” manifestation and difficulties in achieving a scalable intermediate effect.

The ultimate goal of these efforts is to treat human conditions having a neuroinflammatory component with Sx_C_^−^ inhibitors—if not alone, then as part of a combinatorial therapy. Several clinical trials in humans, which involved potential therapeutic agents have failed (especially in stroke), so this novel strategy seems worth investigating. The compounds identified here already serve as convenient molecular probes applicable in research on Sx_C_^−^ transport. Furthermore, the results of this study will guide the development of related optimized molecular tools useful for exploration of the Sx_C_^−^ pharmacophore. Neuroinflammation treatment in vivo studies utilizing a mouse model are in progress.

## Data Availability

This study produced no data sets other than those presented in the figures.
